# Pathways to Positive Youth Development in Malaysian Undergraduate Co-curricular Programs: A Moderated Mediation Model of Youth Voice and Psychological Hardiness

**DOI:** 10.3389/fpsyg.2022.886911

**Published:** 2022-07-14

**Authors:** Katayoun Mehdinezhad Nouri, Steven E. Krauss, Seyedali Ahrari, Ismi Arif Ismail, Mohd Mursyid Arshad

**Affiliations:** ^1^Institute for Social Science Studies, Universiti Putra Malaysia, Serdang, Malaysia; ^2^Faculty of Educational Studies, Universiti Putra Malaysia, Serdang, Malaysia

**Keywords:** positive youth development, youth voice, hardiness, emerging adults, co-curricular programs, Malaysia

## Abstract

Youth voice is gaining more attention globally as a core feature of program quality within positive youth development programs. Few studies have examined the relationship between youth voice and positive youth development in high power-distance cultures, however, where young people often face psychological barriers to exercising decision-making in their work with program adults. Research is needed on the psychological mechanisms that might help youth thrive within settings that are less structurally and psychologically supportive of youth voice. Drawing on bioecological systems and hardiness theories, this quantitative correlational study evaluates the moderating effect of psychological hardiness on the relationship between youth voice, the mediators of program safety and engagement, and the 6 C’s of positive youth development. A moderated mediation model was tested among 436 first-year undergraduate co-curricular program participants from public universities in Malaysia (*M* = 21.192 years, *SD* = 1.191 years; 65.6% female). Youth voice positively predicted positive youth development; the relationship was partially mediated by program engagement, but not safety. The mediated pathway through program engagement was more predictive for hardier youth. By combining programmatic and individual psychological factors into the hypothesized model, this research identifies the potential importance of hardiness on the practice of youth voice for young adults in high power distance cultures. The findings highlight the need to identify other individual and programmatic factors that may contribute to the development of positive youth development in diverse cultural settings.

## Introduction

Positive youth development programs for emerging adults often incorporate a collaborative approach to programming between youth and adults. These relationships are predicated on effective partnership, where the two parties engage in shared decision-making to maximize youth voice and mattering (Schmidt et al., 2020). In both educational and community settings, youth voice has been linked to multiple developmental outcomes including psychological agency, increased self-confidence and empowerment, community connectedness, and critical social and leadership skills (Fox et al., 2008; Zeldin et al., 2017). While research shows strong associations between youth voice and positive youth development, scholars have also noted important barriers to young people’s practice of voice. Fear of adults, lack of prior experience, adult assumptions about youth abilities, role uncertainties and adult-dominated power and control of program settings can hinder young people’s ability to meaningfully participate in decision-making and thus benefit developmentally from their participation in programs (Mitra, 2004; Camino, 2005; Tarifa et al., 2009).

While past research suggests that youth voice fosters youth development, young people’s experiences within culturally diverse populations remains unclear (Ramey et al., 2018). For one, barriers to voice may be intensified in cultures characterized by high-power distance orientation. Scholars have reported that young people from high power-distance cultures are less likely to feel engaged in youth program activities and receive support for exercising voice in decision-making from adults (Sun and Shek, 2012; Umar et al., 2021). Consequently, as more countries embrace youth voice as a core principle of their youth development policy and practice frameworks (Wiium and Dimitrova, 2019), there is a need for more research on the psychological mechanisms that might help youth thrive within settings that are less structurally and psychologically supportive of youth voice. Psychological hardiness is one such construct that continues to gain considerable attention from scholars.

Recent studies from multiple disciplines have shown that “hardier” young people who perceive socially challenging environments more positively and manageable are better able to thrive (Maddi et al., 2013; Kravchuk, 2018; Fominova et al., 2020; McDonald et al., 2022). Hardy attitudes help young people interpret stressful situations as opportunities for learning and growth, and as problems that can be solved by one’s own agency (Abdollahi et al., 2015). Research is needed to examine whether hardier attitudes among youth are associated with greater developmental benefits in cultural settings where youth are less habituated to the practice of voice in decision-making. Drawing on bioecological and hardiness theories, the current study tests whether students who demonstrate stronger attitudes of hardiness experience greater positive youth development from the practice of youth voice. The study also builds on past empirical research by examining the indirect effects of youth voice on positive youth development by way of two key elements of program quality. Program engagement and perceived sense of safety have been consistently linked to the achievement of developmental outcomes resulting from youth voice initiatives (Hirsch, 2005; Dawes and Larson, 2011; Zeldin et al., 2016).

The current study has two main objectives: (1) to test pathways between youth voice, program quality (sense of safety, program engagement) and positive youth development among a sample of first-year Malaysian public university students; and (2) to examine the moderating role of hardiness on these relationships. We hypothesize that students who experience more positive experiences of youth voice will indicate stronger associations with positive youth development, and that these relationships will be mediated by two aspects of program quality—safety and program engagement. We further hypothesize that the pathways of youth voice to positive youth development will be more robust for students who demonstrate greater hardiness.

### Youth Voice and Positive Youth Development

Positive youth development is a strengths-based approach to working with youth that prioritizes youth’s internal and external strengths as indicators of thriving, well-being and the attainment of positive behavioral outcomes (Benson et al., 2012). Positive youth development interventions aim to support adolescents toward the acquisition of a sense of competence, self-efficacy, belonging and empowerment, thereby promoting positive behaviors and reducing the likelihood of participation in high-risk behaviors (Ciocanel et al., 2017). The five Cs (Lerner et al., 2003) act as a guiding framework for describing positive developmental outcomes. The five Cs (i.e., confidence, competence, character, connection, caring) act as universally-desirable characteristics for youth to possess, which, if achieved, lead to the attainment of the sixth C—contribution. The five Cs were derived from extensive reviews of the adolescent development literature and have been linked to a variety of positive program outcomes (Dvorsky et al., 2019). Research suggests that by developing the five Cs, youth are more likely to be active contributors to society and community (Lerner et al., 2003; Fraser-Thomas et al., 2005).

Positive youth development is optimized when youth are engaged in decision-making, particularly when their ideas are considered and respected by supportive adults (Benson et al., 2012; Duke, 2014; Zeldin et al., 2017). Creating opportunities for youth to develop their own voice is a long-established emphasis in youth development practice (Arnold, 2020). Studies on youth voice have shown that the practice promotes a sense of agency, confidence, self-efficacy, empowerment, as well as community connections among youth (Serido et al., 2011; Akiva et al., 2014; Krauss et al., 2014; Ramey et al., 2018). Youth also gain valuable skills, including social and leadership skills, from being included in decision-making processes (Fox et al., 2008). Qualitative studies on the influence of youth voice in communities have noted increased self-confidence to attain personal aims (Zeldin et al., 2005; Mitra, 2008), engagement in programs (Wong et al., 2010), and improved relations with adults (Krauss et al., 2015). Active youth participation on behalf of self and others has been shown to contribute positively to feelings of community attachment, membership, civic identity, and social trust (Youniss et al., 1997; Jarrett et al., 2005; Flanagan et al., 2010). In a related study in Malaysia, youth voice was conceptualized as an important part of the opportunity role structure of youth-led associations and was positively associated with organizational engagement (Krauss et al., 2020).

To date, much of the work examining the links between youth voice and developmental outcomes in out-of-school settings has been limited to school-aged youth and adolescents (Ramey et al., 2018). In tertiary educational settings, student voice has been a concern of scholars for many years, particularly in the areas of student consultation, participation, collaboration, leadership and intergenerational learning (Lee and Wong, 2019). Research on voice at the university level has mostly been descriptive rather than evaluative, however, and for the broad purposes of quality enhancement and assurance of academic programs, staff professional development, and student engagement (Seale, 2009). Less emphasis has been given to examining the developmental contributions of voice in decision-making on student personal growth in co-curricular settings.

#### Program Engagement and Sense of Safety as Mediators

While studies on youth voice and positive youth development have uncovered important linkages, the mechanisms through which this occurs have received considerably less attention. Extant theory and a mostly qualitative literature indicate that when youth experience high degrees of voice, whether in school or community-based program settings, they are more likely to be engaged in their environment and feel safe, thus resulting in developmental rewards (Ramey et al., 2018). The role of program engagement and feelings of safety thus speak to the potential mediating role of program quality. In a study of high school-aged youth in afterschool programs in Malaysia, Zeldin et al. (2016) found that program engagement and feelings of safety mediated the relationships between youth voice and developmental outcomes related to empowerment and community connectedness. When youth perceived more opportunities for voice in their programs, they felt more engaged in programs, which resulted in greater perceived policy control and leadership competence. The results were consistent with earlier qualitative studies suggesting that youth-directed activity engenders feelings of engagement and safety (Hirsch, 2005; Perkins et al., 2007). Strobel et al. (2008) and Whitlock (2007) showed that safety and trust are not only products of active care and concern by adults, but also result when youth have high perceived levels of efficacy in making important choices about their programs. When youth feel that their views and opinions are respected by adults, youth are more inclined to feel that they belong (Mitra, 2004).

#### The Moderating Role of Hardiness

The last two decades have seen an increased interest in hardiness among youth. Hardiness has been conceptualized according to three intersecting dispositions or attitudes: *commitment*—the belief that participation is more useful than disengagement; *challenge*—persistence in learning from experience; where the individual believes that more can be accomplished by engaging in a potentially difficult activity than expecting safety and comfort; and *control*—the belief that struggling to affect outcomes is more beneficial than being passive (Abdollahi et al., 2015). Hardiness enables a person to have a sense of commitment, see life’s difficulties as positive challenges, and have the ability to control one’s emotions when faced with hardship (Maddi et al., 2013). Due to its relationship with problem-solving and coping within challenging social settings, earlier research on hardiness in schools found that hardier students were better able to cope with classroom stressors and overcome threats to academic success (Digman, 1989; Wentzel and McNamara, 1999). More recent studies have shown that hardiness helps young people control stress (Vidrine et al., 2013) and develop problem-solving skills (Abdollahi et al., 2018b), and has been associated with resiliency (Kermani and Mahani, 2015), mental health and behavioral adjustment (Huey Jr and Weisz, 1997; Cumberland-Li et al., 2004), and well-being (Rizvi, 2016). Individuals reported to be higher on measures of hardiness have also been linked to greater levels of happiness, life satisfaction, and mental and physical health (Abdollahi et al., 2015).

Here, the potential nexus between hardiness and youth voice comes into focus. The extant literature points to the potential buffering effect of hardiness on barriers to exercising voice in program settings. Previous studies conducted mostly in Western countries cite lack of respect by adults, youth fear of adults, inadequate training, lack of prior involvement in programs, and adult-dominated power and control of programs as common barriers to voice (Mitra, 2004; Fox et al., 2008; Collins et al., 2016). These challenges can be even more pronounced in high power-distance cultures. A recent qualitative study on young people’s experiences in Nigerian school-based management committees (Umar et al., 2021) identified several socio-cultural factors that limited the ability of young people to play active roles in committee decision-making, including young people’s fear of challenging traditional localized power structures, and a general lack of a participatory culture. Hardiness is gaining traction among scholars, however, as a malleable personal quality that young people can acquire to help them navigate challenging program settings. Ngai et al.’s (2008) study on low-income adolescents in Hong Kong reported that participation in community service programs and hardiness combined to predict two developmental outcomes—work achievement and overall accomplishment. In the same study, hardiness alone further predicted mental health and behavioral adjustment among the sample.

### Theoretical Foundations

To frame our inquiry, the current study draws on bioecological systems (Bronfenbrenner, 2005) and hardiness theories (Kobasa et al., 1982). Bioecological systems theory posits that development occurs through interactions between a youth and his or her environment over time. The influence of these interactions varies based on characteristics of both the youth and the environment (Williams and Deutsch, 2016). The macro-system level of Bronfenbrenner (1977) ecological framework is depicted as the culture in which the individual is embedded, comprised of the cultural values, laws, and customs that influence the micro-, meso-, and exo-systems (Algood et al., 2013). While youth development programs influence development at the micro system level, more distal aspects of the macro system like culturally normative power-distance orientation between youth and adults can indirectly impact youth development. In the case of countries with high power-distance, this often occurs through role expectations and working relationships between youth and their adult program leaders. Adults’ perceived higher position in the society leads them to view youth as less experienced, capable and competent. This perception, in turn, hinders adults’ ability to value youths’ views and program inputs (Hofstede, 2001; Punnahitanond, 2005). Youth, on the other hand, often avoid directly engaging with adult leaders out of fear of being seen as challenging adults’ authority (Chan et al., 2016). The resulting disconnect with adult program staff results in youth not being able to benefit from adult guidance, knowledge and wisdom. It can also reduce both parties’ motivation to participate actively, thereby reducing the program’s influence on youth development (Williams and Deutsch, 2016).

To offset these challenges, hardiness theory stresses that hardier youth will be more actively engaged in program activities by maintaining attitudes that are aligned with a sense of responsibility, caring, and engagement. As such, hardier individuals tend to be better able to control and manage feelings, which enables them to comprehend stressful events as challenges rather than as threats (Florian et al., 1995). A hardy psychological disposition can directly influence a young person’s program interactions by making them more inclined to embrace opportunities to practice voice, even when adults are less forthcoming in providing opportunities to do so. An important part of this process is transformational coping—a way of interpreting stressful life circumstances and social encounters as problems that can be solved by one’s own agency (Maddi and Kobasa, 1984). In the case of a high power-distance cultural setting, a hardier young person—while acknowledging the existence of power-distance with an adult leader—might see such a challenge as an opportunity for growth and positive challenge, rather than a situation to be feared. Hardy coping contrasts sharply to addressing stressors through a regressive coping approach (Maddi and Kobasa, 1984) of denying and avoiding, which tends to be common in collectivist cultures characterized by sharp role distinctions between youth and adults and deference to authority figures (Cortina et al., 2017; Umar et al., 2017). Therefore, youth who have adopted hardier attitudes might perceive the need to practice voice in the face of challenging role expectations as an opportunity for growth, thus reducing the inclination for regressive coping and disengagement.

### The Current Study

In the current study, we posit co-curricular positive development programs as important proximal settings influenced by the larger macro system of Malaysian culture and its high power-distance orientation. Malaysia has been documented as having the highest recorded Power Distance Index of any country in organizational contexts (Hofstede, 2015). In Malaysia, young people retain high levels of respect for elders and those in higher social positions with an emphasis on group orientation and face-saving. This translates over to educational settings, including tertiary settings where the power distance between lecturers and students is considered high (Ismail and Alexander, 2005; Chan et al., 2016). However, Malaysia also has a longstanding commitment to youth development and organized youth programs. Recent national youth policy initiatives have even identified youth participation as a practice priority, situating youth voice as major thrust within youth development programs (Ministry of Education Malaysia, 2015). This presents a unique challenge for Malaysian youth program settings where there is policy support for voice on one hand, yet culturally embedded power distance on the other (Krauss et al., 2020).

Malaysian universities give considerable attention to co-curricular programs targeting positive developmental outcomes such as innovative problem-solving abilities, critical thinking, lifelong learning, ethical leadership, and effective communication. Within these programs, students are expected to exercise voice through assuming significant leadership, critical thinking and decision-making roles in the process of carrying out campus and community-based projects and programs [Pusat Kokurikulum dan Pembangunan Pelajar Center for Co-curriculum and Student, D, 2021]. Many first-year students have little prior experience practicing youth voice, however, due to a lack of authentic participatory experiences during their primary and secondary-school years. In Malaysian secondary schools, for instance, researchers have pointed out that students’ experiences are rarely solicited by adults (Lee and Wong, 2019), while out-of-school-time youth programs for adolescents tend to be highly adult-driven (Nga, 2009; Abdul Rahim, 2020). This lack of experience in practicing voice in the adolescent years can detract from students’ feelings of safety and engagement in co-curricular programs (Krauss et al., 2020).

**Figure 1:**
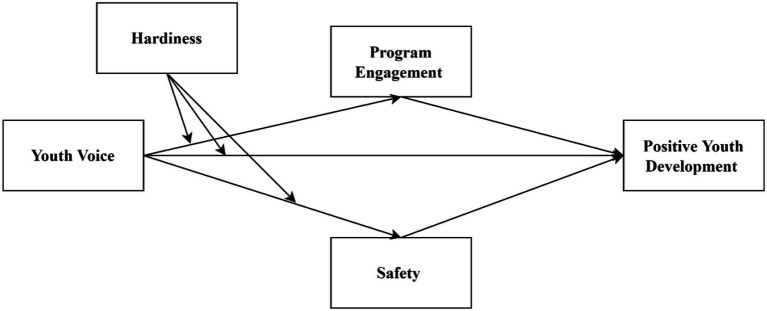
Hypothesized path model.

To conduct the study, we specified a moderated mediation path model that situates youth voice as a predictor that will have direct effects on positive youth development. The model further situates program engagement and safety as psychological mechanisms that are hypothesized to mediate the relationship between youth voice and positive youth development. We predicted the experience of youth voice to have a direct effect on positive youth development (Hypothesis 1). Past research suggests the mediating influences of program engagement and sense of safety on this association. We further hypothesized, therefore, that program engagement and safety would mediate the association between youth voice and positive youth development (Hypothesis 2_a, b_), and that the hypothesized pathways between youth voice and the mediators would be stronger for youth displaying higher levels of hardiness (Hypotheses 4_a, b_; Figure 1). The specific hypotheses of the study were:

*Hypothesis 1*: There is a positive relationship between youth voice and positive youth development.

*Hypothesis 2a*: Program engagement mediates the relationship between youth voice and positive youth development.

*Hypothesis 2b*: Safety mediates the relationship between youth voice and positive youth development.

*Hypothesis 3*: Hardiness moderates the relationship between youth voice and positive youth development.

*Hypothesis 4a*: Program engagement mediates the relationship between youth voice × hardiness and positive youth development.

*Hypothesis 4b*: Safety mediates the relationship between youth voice × hardiness and positive youth development.

It is important to note that causal inferences about mediation should not be made with cross-sectional data (O’Laughlin et al., 2018; Edwards and Konold, 2020), as cross-sectional data sets do not meet the assumption of temporal precedence (Maxwell and Cole, 2007; Little, 2013). Nevertheless, cross-sectional mediation models are used extensively in the social sciences due to their valuable theoretical contributions and exploratory value (Disabato, 2016; Winer et al., 2016). To avoid making causal inferences, we were careful to avoid using causal language in framing our hypotheses and discussing our findings.

## Materials and Methods

### Participants and Procedures

The study sample included 436 first-year undergraduate students from all four public universities in Selangor state [Universiti Putra Malaysia (UPM), Universiti Kebangsaan Malaysia (UKM), Universiti Teknologi Mara (UITM), and Universiti Islam Antarabangsa Malaysia (UIAM)]. Selangor State was chosen due to it being the most populated and racially diverse state in Malaysia with 6.57 million residents and the largest percentage of the overall population of the country (Department of Statistics Malaysia, 2021). Selangor’s and diversity provided the best opportunity for generalizing the study findings. Within the four universities, nine types of co-curricular programs were offered, namely, sports, arts, community service, entrepreneurship, culture, volunteering, innovation and invention, leadership, and public speaking. Approval to carry out the study was first granted by the ethics review committee of the host university, followed by the respective Student Affairs Divisions of each of the universities under study.

According to the Malaysian Ministry of Higher Education, university-based co-curricular programs are compulsory for all first-year undergraduate students [Pusat Kokurikulum dan Pembangunan Pelajar Center for Co-curriculum and Student, D, 2021]. These programs aim to enhance life and leadership skills among students by complementing core curricular activities. The targeted skills include self-discipline, organizational skills, teamwork and cooperation and community engagement (Musa et al., 2016). The programs selected for the current study met four criteria. First, all were developmental in design, focusing on skill and competency building in the areas of entrepreneurship and innovation, sports, arts and culture, community service, public speaking, and leadership. Second, in line with university rules, the programs were regularly carried out at a fixed location (e.g., student halls inside the universities). Third, the programs were headed by college Fellows (adult program staff) who were consistently involved with undergraduate students to allow for relationship-building over time. Finally, all the programs incorporated opportunities for voice in decision-making through direct planning of programs in the form of tournaments, leadership training courses, public relations campaigns, charity and voluntary events (e.g., flood relief), and community education programs (e.g., Dengue Fever awareness and community mobilization programs).

A quantitative correlational study design was employed to answer the research objectives. A multistage, cluster random sampling method was used to carry out data collection. Following selection of the four public universities, four programs (arts, community service, sports, and leadership) were randomly selected. Using random number generator software, 125 students from the selected programs at each university were then randomly selected. Inclusion criteria for the study were that students had to be enrolled as first-year, full-time undergraduate students between the ages of 19 to 24 years old and enrolled in one of the above mentioned co-curricular programs. Questionnaires were distributed among the students at each of the universities, of which 36 (7.2%) were incomplete giving an overall response rate of 92.8%. Another 28 (5.6%) were removed from the analysis due to outlier values. The age range of the sample was between 19 to 24 years (*M* = 21.19, *SD* = 1.19; 65.6% female). Ethnically, the sample closely mirrored Malaysia’s general population as 57.3% of the respondents were Malay, 25.9% were Chinese, 9.2% were Indian, and 7.6% identified as ‘other.’ Respondents reported spending a mean of 9.32 h per week on campus outside of class time, and 6.88 h per week working with program staff.

The survey instrument was originally developed in English and translated to Malay. Three of the five measures were previously translated into Malay and validated in studies with Malaysian youth. To translate the other two instruments and ensure that the items were culturally appropriate for the study sample, items were initially translated and back translated by a Research Officer in Universiti Putra Malaysia with expertise in survey translation. The translation was subsequently checked by two members of the research team, both PhD-level researchers fluent in both English and Malay. During the translation process, the team gave due consideration to the culture of the target population and their age. After agreement was reached on wording, a pilot test was conducted with 50 undergraduate students. Cronbach alpha scores for all measures on the pilot test ranged between 0.718 and 0.962. No further modifications were required. On the final survey, all items were presented in both Malay and English.

The data were collected in person in the Fall of 2019 (prior to the COVID-19 lockdown in Malaysia) by the main author and a research assistant over a period of 3 months. During data collection, the students were encouraged to answer all questions on the survey. They were further informed that their participation was voluntary and that their identities would be kept anonymous. Written informed consent was obtained from the respondents for participation in the study.

### Measures

#### Positive Youth Development

The Six C’s of the Positive Youth Development Inventory (Arnold and Gifford, 2014) was used in this study to assess the positive youth development. The Positive Youth Development Inventory comprises 55 items designed to measure changes in the level of overall positive youth development (Competence—14 items, “I make good decisions,” “I make friends easily”; Character—9 items, “I think it is important for me to be a role model for others,” “It is important for me to do my best”; Connection—8 items, “I have a wide circle of friends,” “I think it is important to be involved with other people”; Caring—8 items, “When there is a need I help whenever I can,” “It is easy for me to consider the feelings of others”; Confidence—9 items, “I general, I think I am a worthy person,” “I know how to behave well in different settings”; and Contribution—7 items, “I take an active role in my community,” “I am someone who gives to benefit others”). The total summed score of the scale was used to determine positive youth development among the study sample. Each item was rated on a 5-point Likert-type scale ranging from 1 = *Strongly Disagree* to 5 = *Strongly Agree*. The Cronbach alpha score for the current study was 0.91.

#### Youth Voice in Decision-Making

The measure of student voice in decision making was adapted from Zeldin et al. (2014). This validation study included a Malaysian sample, so minimal adaptations were required. Participants responded to five statements (e.g., “In this program, I get to make decisions about the things I want to do,” “I have a say in planning programs at this centre”) that were rated using a 5-point Likert-type scale ranging from 1 = *Strongly Disagree* to 5 = *Strongly Agree*. The Cronbach alpha score for the measure was 0.90.

#### Program Engagement

The measure of program engagement, understood as the simultaneous experience of concentration, interest and enjoyment, was adapted from the Vandell et al.’s (2005) study of engagement in youth programs. The adapted measure for program engagement was previously validated with Malaysian secondary school-aged students (Zeldin et al., 2014), therefore, no further adaptations were required. Five statements (e.g., “I find this program to be challenging,” “This program is important to my life”) were rated using a 5-point Likert-type scale ranging from 1 = *Strongly Disagree* to 5 = *Strongly Agree*. The Cronbach alpha score for the current study was 0.91.

#### Sense of Safety

The measure for sense of safety was adapted from the emotional safety rubric from the Youth Program Quality Assessment (Forum for Youth Investment, 2012). The measure was also adapted to assess young people’s sense of safety in program settings. The measure was previously validated among Malaysian secondary-school aged youth in two separate studies (Zeldin et al., 2014; Krauss et al., 2020). Four statements (e.g., “I feel safe when I’m in this program,” “This program makes me feel welcome,” “Bullying and aggression are not tolerated here”) were rated using a 5-point Likert-type scale from 1 = *Strongly Disagree* to 5 = *Strongly Agree*. The Cronbach alpha score for the current study was 0.79.

#### Hardiness

The revised Personal Views Survey (third edition; Maddi et al., 2006) consists of 18 positively and negatively worded statements that evaluate the three components of hardiness—control, commitment and challenge. Past scholarship on hardiness cautions against separating the hardiness components of commitment, control, and challenge for the sake of measurement (Maddi et al., 2006). Therefore, the sum of the three scores was used. Sample items for each include Control (“By working hard, you can always achieve your goal,” “I do not like to make changes in my everyday schedule”), Commitment (“I really look forward to my work,” “No matter how hard I try, my efforts usually accomplish little”), and Challenge (“I am not equipped to handle the unexpected problems of life,” “I like a lot of variety in my work”). The Personal Views Survey has been found to be of satisfactory reliability and validity among Malaysian undergraduate students (Abdollahi et al., 2015, 2018b). A 4-point Likert-like scale was used for all items ranging from 0 = *Not at All True* to 3 = *Very True*. Previous studies have shown an acceptable level of internal consistency (test–retest coefficient, interval between 2 and 4 weeks) for overall hardiness (Cronbach α—0.80 to 0.88; Abdollahi et al., 2018a). The Cronbach alpha score for the current study was 0.89.

### Analytic Strategy

Partial least squares structural equation modeling (PLS-SEM) was used to validate the research model (Kock, 2017). We analyzed the data using the partial least squares algorithm with a bootstrapping set at 5,000 subsamples using Smart-PLS 3.3.3 software (Hair Jr et al., 2020). Partial least squares structural equation modeling was chosen above other regression models because it is adept at handling the study’s complex explanatory model, and the objective of the study is exploratory in nature rather than theory confirming (Hair et al., 2017; Henseler et al., 2018; Ringle et al., 2020).

Statistical Package for the Social Sciences (SPSS, version 26) software was used to estimate the quantity of missing data, which was found to be less than 2%. The missing data were then addressed using the regression imputation approach (Kock, 2018). The moderating function of hardiness in the youth voice-positive youth development association was investigated using the interaction-moderation approach. To calculate the standard error fort-value computation, the researchers used a bootstrapping method. Confidence intervals that do not include zero have means effects that are significant at *α* = 0.05. Model fit was assessed using the Standardized Root Mean Square Residual (SRMR) and the Bentler-Bonett Normed Fit Index (NFI). The differences between observed and expected correlations were evaluated by the SRMR, while the incremental measure of goodness of model fit was represented by the NFI.

#### Testing for Moderated Mediation

We assessed if a mediational process was conditional on other factors using moderated mediation (Hayes, 2017). In hypotheses 4a and 4b, we assumed that hardiness moderates the association between youth voice and positive youth development. As such, we examined the possibility that hardiness may provide a conditional influence on the strength of the indirect relationship between youth voice and positive youth development through program engagement and safety. We predicted a strong (weak) relationship between youth voice and program engagement and youth voice and safety when hardiness is high (low).

## Results

### Preliminary Analysis

Table 1 lists the means, standard deviations, Cronbach alphas and bivariate correlations for the study variables. The skewness and kurtosis of all variables were within the acceptable ranges. As expected, youth voice was positively correlated with positive youth development, sense of safety, program engagement and hardiness. To check the properties of the measurement scales, we assessed reliability, convergent validity, and discriminant validity of the scales. First, factor loading values for all measures were above 0.70, indicating that each research variable achieved acceptable convergent validity. All composite reliability (CR), Cronbach alpha (CA) estimates and average variance extracted (AVE) values were above their cut off values of 0.7 and 0.5, respectively (Hair et al., 2016; Henseler et al., 2016).

**Table 1:** Descriptive statistics and bivariate correlations.

Variable	M	SD	α	1	2	3	4	5
Positive youth development	3.853	0.509	0.91	1				
Youth voice	3.755	0.671	0.90	0.808^**^	1			
Program engagement	3.683	0.634	0.91	0.722^**^	0.657^**^	1		
Safety	3.912	0.603	0.79	0.560^**^	0.601^**^	0.567^**^	1	
Hardiness	1.603	0.561	0.89	0.457^**^	0.402^**^	0.351^**^	0.314^**^	1

*N* = 436.

** *p* < 0.01.

Discriminant validity was tested using the Fornell-Larcker and Heterotrait-Monotrait (HTMT) criteria as recommended by Fornell and Larcker (1981) and Henseler et al. (2015). For the Fornell-Larcker criterion, the results indicated that the square root of each construct’s AVE was higher than the correlation values of the other constructs. The Heterotrait-Monotrait (HTMT) analysis showed that the results were within the recommended threshold of <0.85 (Franke and Sarstedt, 2019). The findings confirm that there was mutual discrimination between the study measures (Table 2).

**Table 2:** Measurement model: discriminant validity.

	Fornell-Larcker Criterion					Heterotrait-Monotrait Ratio (HTMT)			
	1	2	3	4	5	1	2	3	4
1. HAR	0.752								
2. PYD	0.452	0.825				0.498			
3. SE	0.305	0.572	0.783			0.359	0.667		
4. PE	0.359	0.729	0.581	0.827		0.393	0.803	0.674	
5. YV	0.393	0.808	0.61	0.659	0.842	0.438	0.846	0.715	0.729

*YV = youth voice; HAR = hardiness; PE = program engagement; SE = safety; PYD = positive youth development*.

### Hypothesis Testing

#### Structural Model

We used path analysis to evaluate H_1_. The structural model was assessed through analysis of the path coefficients, coefficient of determination (R^2^), and predictive relevance (Q^2^). The model was tested by conducting a nonparametric bootstrapping procedure with a resample of 5,000 to generate the *β* and corresponding *t*-values. Results confirmed that the data fit the model well as SRMR values of all models were less than 0.051 and NFI values were greater than 0.88 (Henseler et al., 2016). R^2^ was calculated to estimate the variance value in positive youth development *via* youth voice. The R^2^ for positive youth development was 0.654, indicating a moderate-to-substantial relationship (Henseler et al., 2015). Collinearity was assessed by calculating the VIF values, which were less than five for all constructs in the analysis, indicating that collinearity was not a threat.

Q^2^ was calculated to determine the predictive relevance of positive youth development. The Q^2^ result of 0.415 indicated that positive youth development achieved a large predictive relevance (Henseler et al., 2015). The results further showed a significant positive association between youth voice and positive youth development (*β* = 0.808, *t* = 48.873, *p* < 0.001). Thus, H_1_ was supported.

#### Mediation Tests of Program Engagement and Safety

We used a bootstrapping procedure to examine the proposed mediation model (Preacher and Hayes, 2008). A sample of 5,000 was taken from the original data by random sampling with replacement to assess the indirect relationship between youth voice on positive youth development mediated by program engagement and safety. As shown in Table 3, the indirect effect of youth voice on positive youth development was *β* = 0.539, *t* = 3.294 (*p* < 0.001). To test the parallel mediation, the bootstrapping technique was used by conducting the resampling procedure with a substitution, which has insignificant characteristics to the normality distribution of data (Preacher and Hayes, 2008). In the presence of the mediators, the direct effect between youth voice and positive youth development was significant (*β* = 0.742, *t* = 33.663, *p* < 0.001). For the indirect effects, program engagement partially mediated the relationship between youth voice and positive youth development (*β* = 0.191, *t* = 7.503, *p* < 0.001), while sense of safety did not (*β* = 0.012, *t* = 0.583, *p* = 0.059). Therefore, H_2a_ was supported and H_2b_ was not.

**Table 3:** Mediation effects of program engagement and safety on relationship between youth voice and positive youth development.

**Model 1**			**Model 2**			**Model 3**		
**Total Effect**			**Direct Effect**			**Indirect Effect**		
**Path**	***β***	***t***	**Path**	***β***	***t***	**Path**	***β***	***t***
YV→PYD	0.539^***^	3.294	YV→PYD	0.742^***^	33.663	YV→PE→PYD	0.191^***^	7.503
						YV→SE→PYD	0.012	0.583

*N* = 436; YV = youth voice; HAR = hardiness; PE = program engagement; SE = safety; PYD = positive youth development.

*** *p* < 0.001.

#### Moderating Effect of Hardiness

The interaction-moderation method in Smart-PLS 3 software was used to test the moderating role of hardiness on the link between youth voice and positive youth development. The results indicated significant relationships between youth voice and positive youth development (*β* = 0.746, *t* = 32.519, *p* < 0.001), and between hardiness and positive youth development (*β* = 0.16, *t* = 4.552, *p* < 0.001). The interaction between hardiness and youth voice had a positive and significant relationship with positive youth development (*β* = 0.063, *t* = 2.334, *p* = 0.026), indicating that hardiness played a moderating role in the link between youth voice and positive youth development. Thus, H_3_ was supported (Table 4).

**Table 4:** Moderated mediation effects of program engagement and safety on positive youth development.

**Predictors**	**Model 1 (PE)**		**Predictors**	**Model 2 (SE)**	
	***β***	***t***		***β***	***t***
YV	0.539^***^	16.042	YV	0.539^***^	16.042
HAR	0.16	4.552	HAR	0.16	4.552
YV × HAR	0.087^***^	2.645	YV × HAR	0.057	1.258
PE	0.191^***^	7.503	SE	0.012	0.583
PE × HAR	0.027^**^	2.395	SE × HAR	0.001	0.382
R^2^	0.45		R^2^	0.377	
F	3.128		F	0.345	

*N* = 436; YV = youth voice; HAR = hardiness; PE = program engagement; SE = safety; PYD = positive youth development.

*** *p* < 0.001;

** *p* < 0.01.

#### Moderated Mediating Effect

**Figure 2:**
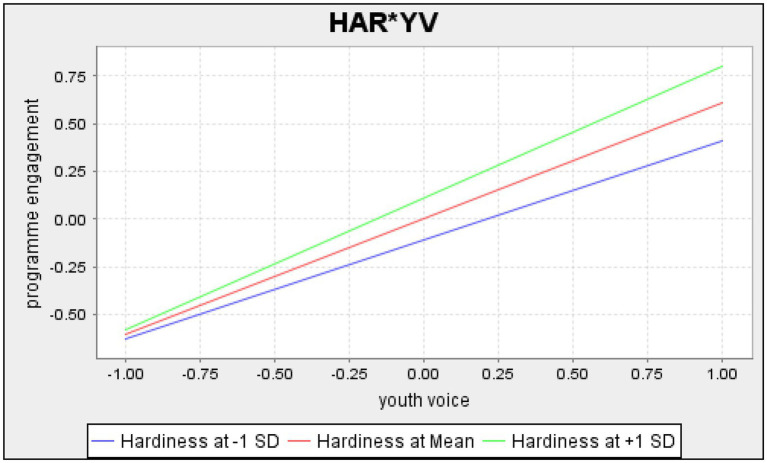
Results of the moderating effect of hardiness on the relationship between youth voice and program engagement.

When a moderation relationship is present, in this case between the indirect effect of youth voice on positive youth development in the presence of program engagement and safety, moderated mediation is applied (Hayes, 2017). The indirect effect corresponds with the value of hardiness, the moderating variable. We carried out the index of moderated mediation to evaluate the conditional process model (Hayes, 2017), where the index quantifies the linear association between the moderator and the indirect effect. The results indicated that hardiness significantly moderated the indirect effect of youth voice on positive youth development (95% CI: 0.03 to 0.046). Specifically, the indirect effect of youth voice on positive youth development through program engagement was stronger as hardiness increased. Therefore, H_4a_ was supported (Figure 2). In contrast, with the inclusion of zero in the confidence interval (95% CI, −0.004 to 0.008), moderated mediation was not supported for sense of safety. The indirect effect of youth voice on positive youth development through safety did not depend on level of hardiness. Thus, H_4b_ was not supported.

## Discussion

The current study sought to examine pathways through which student voice may influence positive youth development among first-year university students in Malaysia. In line with our study hypotheses and previous scholarship, we found that youth voice positively predicted positive youth development. We further found that the relationship between voice and positive youth development was partially mediated by program engagement, and that the model was more robust for hardier students. Sense of safety did not mediate the relationship between voice and positive youth development. The model containing student voice and program engagement explained nearly half (45%) of the variance in positive youth development, indicating that both variables may be of high importance for the development of positive youth development in Malaysian tertiary co-curricular settings.

A growing body of evidence indicates that youth voice is a critical practice for the successful attainment of positive youth development outcomes. This study replicates these findings in a unique Southeast Asian country context where youth development is prioritized, but cultural practices often present challenges to youth empowerment (Stivens, 2020). In line with past studies on youth-adult partnership in Malaysian afterschool and community-based youth programs (Krauss et al., 2014; Zeldin et al., 2016), youth voice predicted positive youth development and that the relationship was partially mediated when youth’s psychological engagement in program activities was enhanced (Strobel et al., 2008). This finding adds to the growing body of evidence that the practice of youth voice and acting affirmatively on proximal environments can influence developmental outcomes, particularly when young people are able to participate on their own terms and contribute to group decisions (Serido et al., 2011; Ramey et al., 2018). Such experiences can produce heightened enjoyment, intrinsic motivation, a sense of mastery and confidence in youth’s ability to complete tasks (Hansen et al., 2003; Dawes and Larson, 2011).

The results also point to the amplifying effect that hardiness can play for youth attempting to exercise voice in culturally challenging program settings. In line with our study hypothesis and hardiness theory, the indirect effect of youth voice on positive youth development through program engagement was stronger as hardiness increased. Although design limitations prevent us from drawing causal inferences, the results suggest that hardier youth might benefit more from the practice of voice, and that voice was more engaging and more closely associated with positive youth development for hardier youth. This buttresses previous findings among secondary-aged youth that young people who participate in positive youth development programs and who are higher in hardiness are more likely to thrive (Ngai et al., 2008; Neil, 2012). The findings also suggest that hardiness acts as a buffer against the potential for reduced engagement in program activities as a result of high-power distance relationships with program adults, which can result in reduced collaboration in program activities. Hardy young people often display higher levels of social competence and problem-solving skills than those with less hardy attitudes (Rutter, 1989; Seligman, 1990). These skills can be greatly beneficial in helping young people navigate potentially uncomfortable social interactions. Hardier attitudes can act to decrease power distance through viewing the challenge of working with adults positively, as an opportunity to grow and find new avenues of meaning, or as an opportunity for increasing social support and personal growth (Maddi and Harvey, 2006).

In contrast with previous studies, sense of safety did not successfully mediate the relationship between voice and positive youth development. The practice of youth voice has been shown to enhance young people’s sense of safety by engendering feelings of trust and connectedness to others, leading to a sense of inclusivity where youth feel free to share their views and participate openly (VanderVen, 2007). The results showed that even though safety was directly associated with positive youth development, program engagement had a stronger mediating effect for our sample of undergraduate students. This finding resonates with stage-environment fit theory (Eccles et al., 1993), which suggests that engagement will be high when there is an appropriate match between the characteristics of an activity and the developmental needs of the young person (Greene et al., 2013). In this case, the findings point to the developmentally appropriate value late adolescents and early adults place on opportunities to exercise voice, over and above feelings of safety. Few studies have examined these program dynamics among university-age youth. Past studies examining the role of program quality within positive youth development programs have been mostly limited to adolescent populations. However, in many non-WEIRD (Western, Educated, Industrialized, Rich, Democratic) countries, where conceptualizations of youth extend into early adulthood, there is a need to better understand the interplay between program contexts and individual factors that can impact development. The current study contributes to this growing literature.

### Limitations and Directions for Future Research

To our knowledge, this is the first empirical study of emerging adults within an Asian positive youth development program context that examines the amplifying effect of hardiness on the relationship between youth development program quality and positive youth development. Replication and design considerations are warranted to address methodological limitations. As mentioned, the cross-sectional design of the study limited our ability to make causal inferences. Our sampling frame was also limited to public university students from four purposively-selected institutions. Lerner and Chase (2019) elaborate on the lack of longitudinal studies on youth development programs internationally as a limitation detracting from a more robust cross-cultural understanding of positive youth development. Future research grounded in methods capable of identifying specific individual-context relations linked to positive youth development for specific groups and nations is needed. Our mediation model was restricted to two elements of positive youth development program setting quality and omitted others such as supportive adult relations (Larson et al., 2005). Future studies should consider more in-depth analyses of how supportive adults can help youth build up hardiness attitudes over time and within the constellation of program activities. In their study, Ngai et al. (2008) concluded that young people are more likely to develop hardy dispositions *through* their participation in programs. How adults support that process, such as in the form of creating more opportunities to practice voice, solve real-world problems, engage in reflective learning and others, requires further exploration by researchers. The results may also be culturally constrained, and, therefore, replication is needed in other cultures. With the global embrace of positive youth development along with corresponding strengths-based and empowerment-focused practices like youth voice, it is important that researchers from diverse cultural settings continue to explore the different ways that culture shapes positive youth development program settings and outcomes.

The study findings suggest that hardier students are more likely to benefit from active participation in youth programs, despite the challenges they might facing in doing so. Their hardiness helps them to view such experiences as growth-related, therefore enhancing their sense of engagement in the activities. This is an important finding for policy makers in Malaysian university co-curricular programs who, falling back on cultural assumptions about youth, may be reluctant to offer young people authentic opportunities for voice. Along the lines of a critical youth development approach that emphasizes how youth programs can be (re)structured to “challenge dominant social narratives for reimagining a more equitable future for youth” (McGee, 2019, p: 96), the experiences of hardy young people in Malaysian co-curricular programs can be used as a basis for advocacy efforts aimed at reducing barriers to the practice of voice (Ballard et al., 2021). In his qualitative study on Nigerian school-based management committees, Umar (2016) demonstrated that cultural barriers to voice were mitigated through advocating for structural reforms, developing more positive relationships between youth and adults, and training for both youth and adult committee members on the benefits of voice.

At the practice level, the findings speak to the malleability of hardiness as an individual characteristic, and whether it can be acquired by young people, making them more resilient in the face of stressors and challenges. According to Abdollahi et al. (2015), hardiness can be learned through developing effective coping skills, learning to interact effectively with others by giving and taking assistance, support, and encouragement, and learning how to use feedback to improve hardy attitudes. Given the potential of hardiness as a resource for coping with stressful circumstances, policy makers at the institutional and program level should consider building hardiness training into programs themselves, with the aim of strengthening young people’s emotional and behavioral development (Ngai et al., 2008).

## Data Availability Statement

The raw data supporting the conclusions of this article will be made available by the authors, without undue reservation.

## Ethics Statement

The studies involving human participants were reviewed and approved by the ethics committee of Universiti Putra Malaysia (JKUPM; UPM/TNCPI/RMC/1.4.18.2). The patients/participants provided their written informed consent to participate in this study.

## Author Contributions

KN: conceptualization, investigation, data curation, formal analysis, and writing—original draft preparation. SK: conceptualization, methodology, funding acquisition, writing—original draft preparations, and review and editing. SA: formal analysis, visualization, and writing—original draft preparations. II and MA: conceptualization, methodology, supervision, and reviewing. All authors contributed to the article and approved the submitted version.

## Funding

This research was funded by Universiti Putra Malaysia’s Putra Grant scheme (Vote # 9596800).

## Conflict of Interest

The authors declare that the research was conducted in the absence of any commercial or financial relationships that could be construed as a potential conflict of interest.

## Publisher’s Note

All claims expressed in this article are solely those of the authors and do not necessarily represent those of their affiliated organizations, or those of the publisher, the editors and the reviewers. Any product that may be evaluated in this article, or claim that may be made by its manufacturer, is not guaranteed or endorsed by the publisher.
